# Role of Ramadan specific diabetes education (RSDE); A prospective study

**DOI:** 10.12669/pjms.333.12345

**Published:** 2017

**Authors:** Muhammad Yakoob Ahmedani, Shahid Ahsan, Muhammad Saif ul Haque

**Affiliations:** 1 Muhammad Yakoob Ahmedani, FCPS. Professor of Medicine, Department of Medicine, Baqai Institute of Diabetology and Endocrinology, Baqai Medical University, Plot No. 1-2, II-B, Nazimabad No.2, Karachi, Pakistan; 2 Shahid Ahsan, MBBS, M.Phil (Biochemistry), M.Phil (Public Health). Associate Professor, Department of Biochemistry, Hamdard College of Medicine and Dentistry, Hamdard University, Karachi, Pakistan; 3 Muhammad Saif ul Haque, MS (Diab & Endo), Registrar, Department of Medicine, Baqai Institute of Diabetology and Endocrinology, Baqai Medical University, Plot No. 1-2, II-B, Nazimabad No.2, Karachi, Pakistan

**Keywords:** Diabetes, Education, Ramadan

## Abstract

**Objective:**

To observe the role of Ramadan Specific Diabetes Education (RSDE) in the management of fasting patients with diabetes.

**Methods:**

This prospective study was carried out at out-patients department (OPD) of Baqai Institute of Diabetology & Endocrinology (BIDE), in 2012. Recruitment of patients started a month prior to Ramadan. Muslim patients with diabetes whether had their first or on follow up visit to the OPD and showed intention to hold fast in the month of Ramadan, were included. A printed broacher focused on six cardinal areas of fasting and diabetes identified in Ramadan specific guidelines was given to all participants. All patients had their first visit to the OPD (n=32) were also given RSDE on one-to-one basis (Group A). Whereas patients had follow up visit were advised to attend a group session on RSDE. Those attended (n= 25) and those did not opt (n=45) the group session were included in Group B and Group C respectively. All participants were instructed to visit the OPD after Ramadan. Group D was constituted after Ramadan. It included patients who had not visited the OPD during induction period thus did not receive RSDE (n=76) they however hold fast in the month of Ramadan. Data regarding compliance to structured education through different modes was collected during post Ramadan visit.

**Results:**

Comparisons among groups who received education(A with B with C) revealed non-significant difference in self-monitoring of blood glucose, alteration of drug dosage and timing, appreciation of hypoglycemia and action taken on development of hypoglycemic symptoms. However, significant differences were noted when group who received education was compared individually with group who did not receive education.

**Conclusion:**

Patients who receive Pre-Ramadan diabetes education were found to be significantly better in following Ramadan specific diabetes management recommendations compared to patients who did not receive education. Further large scale studies are needed to validate our findings.

## INTRODUCTION

Muslims constitute about 1.66 billion of the world’s population and approximately each year more than 50 million Muslims diabetic patients fast in Ramadan.^1^ Ramadan is the 9^th^ month of Muslim calendar and fasting in Ramadan is one of the five basic pillars of Islam. It is obligatory for all healthy adult Muslim to hold fast from dawn to dusk. Timing of meal changes in Ramadan. Those who hold fasting usually take two meals, one before dawn (Sehar) and other before dusk. Between dusk and dawn there is no restriction in the intake of food or fluid.^2^

Though according to religious rulings, travelers, lactating, pregnant and menstruating women, patients who are very sick or those whose health can be adversely affected by holding fast are exempted. A large number of individuals with diabetes were reported to hold fast during Ramadan. A large multicentered study conducted in 13 Islamic countries reported that 43% and 79% of patients with type-1 and type-2diabetes respectively hold fast during Ramadan. However some of these patients did not consult their physicians at all before Ramadan nor change their drug dosage and timing or monitor their sugar level even when they developed acute complications. The study also reported higher rate of acute complications in fasting patients with diabetes and found out several deficiencies in the care and awareness of these patients.^3^

Studies revealed that nearly 50-80% of people with diabetes showed lack of significant knowledge and skill to manage the condition efficiently.^4^,^5^ Evidence suggested that diabetes education has an overall beneficial impact on health and psychological outcomes.^6^ Similarly, studies found significant impact of Ramadan specific education on the reduction of acute complications of diabetes.^7^,^8^

There are few studies conducted to identify the role of education in the management of diabetes during Ramadan. This study explores the role of education in the management of diabetes for safe fasting during Ramadan.

## METHODS

This study was conducted at the outpatient department of the Baqai Institute of Diabetology and Endocrinology. Study was approved from the institutional Review Board of the BIDE. After obtaining signed informed consent, recruitment of patients for the study started one month before Ramadan in 2012.

### Inclusion Criteria

Subjects with type-1 or type-2 diabetes who showed intention to hold fast were included in the study.

### Exclusion Criteria

Patients with complications like uncontrolled hypertension, unstable angina, chronic kidney disease or renal failure, liver disease, any neurological problem, elderly patients with alertness problem, pregnant women, newly diagnosed diabetics (less than 3-monthduration) or patients with brittle type-I diabetes were excluded from the study.

### Socio demographic and biochemical data

Information regarding age, duration and type of diabetes, BMI, blood pressures (systolic and diastolic), current treatment and co-morbidities were collected from patient at the time of recruitment.

### Education sessions

All study participants were given printed broachers which had information focused on six cardinal areas of fasting and diabetes identified in Ramadan specific guidelines. These include alteration of drug dosage and timing, self-monitoring of blood glucose, avoidance of strenuous physical activity during Ramadan, information about diet and fluid intake, awareness of hyperglycemia and hypoglycemic symptoms and action to be taken in response to these complications. It also included knowledge about when to break the fast. Beside brochure all patients had their first visit to the OPD of BIDE (n=32) were also given RSDE on one-to-one basis (Group A). Whereas patients who had follow up visit were advised to attend a group session on RSDE. Those attended (n= 25) and those did not opt (n=45) the group session were included in Group B and Group C respectively. In both one-to-one and group session investigators ensured delivery of similar information to the participants as printed in the brochure. All participants were instructed to visit the OPD after Ramadan.

Group D was constituted after Ramadan. It included patients who had not visited the OPD of BIDE during induction period but hold fast in the month of Ramadan thus did not receive RSDE (n=76) before Ramadan.

Data was collected after Ramadan on a questionnaire from all participants about their compliance to RSDE. Data was entered and analyze by SPSS version 13.0 and represented as mean (±SD) and n (%). A Chi-square or student t test was used where applicable. A p≤0.05 was considered statistically significant.

## RESULTS

**Fig.1:**
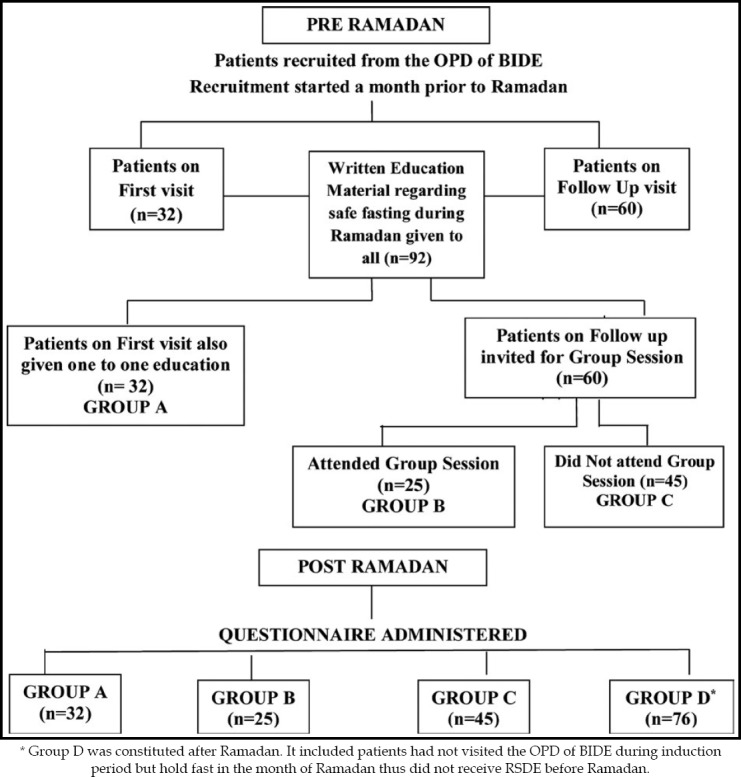
Induction of Study participants.

In the present study, a total of 178 subjects were included. Among them 102 were given education via three different modes namely one to one (n=32), in group (n=25), and through written educational materials (n=45). While, 76 subjects served as control, inducted post-Ramadan in the study (Fig.1).

Baseline characteristics of all the participants received education (A+B+C) and did not receive education (Group D) was shown in the Table-I. Comparison of participants who received education (group A, B and C) with those who did not receive education was shown in Table-II. Significant difference was found in drug dosage and timing before Ramadan, self-monitoring of blood glucose (SMBG) and breaking the fast on experiencing hypoglycemic symptoms. Most of the patients in the education group, in response to hypoglycemic symptoms, checked their blood glucose level and about 20% patients in the education group broke their fast. On development of hypoglycemia, non-significant difference in frequency of hyperglycemic episodes between education and non-education group was reported. Nevertheless, in response to symptoms of hyperglycemia more patients in the education group checked their blood glucose level (Table-II).

**Table-I:** Baseline demographic and clinical characteristics of the study population.

	*Those who receive education (A+B+C)*	*Those who do not receive education (D)*	*Overall*
n	102	76	178
Age (years)	46.2 ± 15.1	49.6 ± 11.8	47.7 ± 13.9
***Gender***			
*Type of Diabetes*			
Type-1	20 (19.6%)	2 (2.6%)	22 (12.4%)
Type-2	82 (80.4%)	74 (97.4%)	156 (87.6%)
Duration of diabetes(years)	9.7 ± 7.4	7.3 ± 6.9	8.6 ± 7.2
Weight (kg)	70.2 ± 15.8	77.5 ± 16.4	73.4 ± 16.4
Height (cm)	160.9 ± 8.8	165.5 ± 8.8	162.9 ± 9.1
Body mass index (kg/m^2^)	27.2 ± 6.2	28.3 ± 5.5	27.7 ± 6.0
*Education Level*			
No formal education	7 (6.9%)	4 (5.3%)	11 (6.2%)
Can read/write	17 (16.7%)	11 (14.5%)	28 (15.7%)
Matric	16 (15.7%)	18 (23.7%)	34 (19.1%)
Intermediate	19 (18.6%)	9 (11.8%)	28 (15.7%)
Graduate	30 (29.4%)	20 (26.3%)	50 (28.1%)
Post graduate	13 (12.7%)	14 (18.4%)	27 (15.2%)
*Occupation*			
Student	10 (9.8%)	1 (1.3%)	11 (6.2%)
House wife	34 (33.3%)	16 (21.1%)	50 (28.1%)
Manual labor	6 (5.9%)	9 (11.8%)	15 (8.4%)
Office worker	25 (24.5%)	18 (23.7%)	43 (24.2%)
Manager/Businessman	18 (17.6%)	22 (28.9%)	40 (22.5%)
Retired/Unemployed	9 (8.8%)	10 (13.2%)	19 (10.7%)
*Type of Treatment*			
OHA	29 (28.4%)	51 (67.1%)	80 (44.9%)
Insulin	41 (40.2%)	8 (10.5%)	49 (27.5%)
OHA & Insulin	32 (31.4%)	17 (22.4%)	49 (27.5%)
*Fasting History*			
Average number of fasts during this Ramadan	27.3 ± 5.7	27.9 ± 5.0	27.6 ± 5.4
Mean history of fasting (years)	31.4 ± 13.7	34.3 ± 10.8	32.6 ± 12.7

***Note:*** Data presented as mean ± s.d or n (%)

**Table-II:** Comparison of groups received Ramadan specific diabetes education (A+B+C) with non-education group (D) on cardinal aspect of diabetes and fasting.

	*Received education (Group A+B+C)*	*Did not receive education (Group D)*	*P-value*	*Overall*
n	102	76	-	178
**Drugs**				
**Changed Drug dosage and timings before Ramadan?**				
Yes	81 (79.4%)	23 (30.3%)	<0.0001	104 (58.4%)
No	21 (20.6%)	53 (69.7%)		74 (41.6%)
***Who change drug dosage and timings?***				
Physician	70 (86.4%)	21 (91.3%)	0.7820	91 (87.5%)
Diabetes educator	4 (4.9%)	1 (4.3%)		5 (4.8%)
Others	7 (8.6%)	1 (4.3%)		8 (7.7%)
**Exercise**				
**Did exercise during Ramadan?**				
Yes	18 (17.6%)	19 (25%)	0.1560	37 (20.8%)
No	84 (82.4%)	57 (75%)		141 (79.2%)
**Type of exercise during Ramadan**				
Light (walking, gardening)	12 (66.7%)	18 (94.7%)	0.1380	30 (81.1%)
Sedentary (house bound)	2 (11.1%)	1 (5.3%)		3 (8.1%)
Moderate(brisk walking, swimming)	3 (16.7%)	0 (0.0%)		3 (8.1%)
Heavy(running, energetic sports)	1 (5.6%)	0 (0.0%)		1 (2.7%)
**Performed SMBG during Ramadan?**				
Yes	96 (94.1%)	50 (65.8%)	<0.0001	146 (82.0%)
No	6 (5.9%)	26 (34.2%)		32 (18.0%)
**Appreciated Symptoms of hypoglycemia?**				
Yes	35 (34.3%)	13 (17.1%)	0.0080	48 (27.0%)
No	67 (65.7%)	63 (82.9%)		130 (73.0%)
**Frequency of symptoms of hypoglycemia**				
Once/month	13 (37.1%)	3 (23.1%)	0.6940	16 (33.3%)
Twice/month	9 (25.7%)	3 (23.1%)		12 (25.0%)
Thrice/month	8 (22.9%)	5 (38.5%)		13 (27.1%)
Others	5 (14.3%)	2 (15.4%)		7 (14.6%)
**Checked blood glucose level if developed symptoms of hypoglycemia?**				
Yes	16 (45.7%)	3 (23.1%)	0.0670	19 (39.6%)
No	14 (40.0%)	7 (53.8%)		21 (43.8%)
Not responded	5 (14.3%)	3 (23.1%)		8 (16.7%)
**If developed symptoms of hypoglycemia**				
Continued fast	27 (77.1%)	11 (84.6%)	0.0800	38 (79.2%)
Broke the fast	7 (20.0%)	0 (0.0%)		7 (14.6%)
Visited doctor	1 (2.9%)	2 (15.4%)		3 (6.3%)
Needed hospitalization	0 (0.0%)	0 (0.0%)		0 (0.0%)
**Appreciated Symptoms of hyperglycemia?**				
Yes	27 (26.5%)	23 (30.3%)	0.3480	50 (28.1%)
No	75 (73.5%)	53 (69.7%)		128 (71.9%)
**Frequency of symptoms of hyperglycemia**				
Once/month	8 (29.6%)	5 (21.7%)	0.2150	13 (26%)
Twice/month	6 (22.2%)	4 (17.4%)		10 (20%)
Thrice/month	2 (7.4%)	7 (30.4%)		9 (18%)
Others	11 (40.7%)	7 (30.4%)		18 (36%)
**Checked blood glucose level if developed symptoms of hyperglycemia?**				
Yes	16 (59.3%)	12 (52.2%)	0.3920	28 (56.0%)
No	6 (22.2%)	7 (30.4%)		13 (26.0%)
Not responded	5 (18.5%)	4 (17.4%)		9 (18.0%)
**If developed symptoms of hyperglycemia**				
Continued fast	18 (66.7%)	12 (52.2%)	0.0920	30 (60.0%)
Broke the fast	0 (0.0%)	1 (4.3%)		1 (2.0%)
Visited doctor	0 (0.0%)	3 (13.0%)		3 (6.0%)
Needed hospitalization	0 (0.0%)	0 (0.0%)		0 (0.0%)
Not responded	9 (33.3%)	7 (30.4%)		16 (32.0%)

***Note:*** Data presented as Mean ± SD; n (%)

Subsequent analysis by comparing the education groups among each other (A with B with C) revealed non-significant difference among groups in self-monitoring of blood glucose, alteration of drug dosage and timing, frequency of hypoglycemia, monitoring of blood glucose and breaking of fast on development of hypoglycemic symptoms. The differences in above variables however attain significance when each group who receive education was compared individually with the group who did not receive education (A or B or C, with D) (Table-III).

**Table-III:** Comparison of groups received Ramadan specific diabetes education among each other and with non-education group (D) on cardinal aspect of diabetes and fasting.

	*Received education*			*Did not receive education*

	*One-to-one Session (Group A)*	*Group session (Group B)*	*Written education material (Group C)*	*Did not receive education (Group D)*
n	32	25	45	76
Performed SMBG during Ramadan?	32 (100.0%)	23 (92.0%)	41 (91.1%)	50 (65.8%)^abc^
Changed drug dosage and timings before Ramadan	25 (78.1%)	22 (88.0%)	34 (75.6%)	23 (30.3%)^abc^
Did Exercise during Ramadan	4 (12.5%)	5 (20.0%)	9 (20.0%)	19 (25.0%)
Appreciated symptoms of hypoglycemia	13 (40.6%)	11 (44.0%)	11 (24.4%)	13 (17.1%)^ab^
Monitored blood glucose if developed symptoms of hypoglycemia	6 (46.1%)	6 (54.5%)	4 (36.3%)	3 (23.1%)^ab^
Broke the fast if developed symptoms of hypoglycemia	2 (15.4%)	2 (18.2%)	3 (27.3%)	0 (0.0%)^abc^
Appreciated symptoms of hyperglycemia	9 (28.1%)	5 (20.0%)	13 (28.9%)	23 (30.3%)
Monitored blood glucose if developed symptoms of hyperglycemia	4 (44.4%)	4 (80.0%)	8 (61.5%)	12 (52.2%)
Broke the fast if developed symptoms of hyperglycemia	0 (0.0%)	0 (0.0%)	0 (0.0%)	1 (4.3%)

***Note:*** Data presented as n (%), * For p < 0.05 One-to-one session vs. Group session, † For p < 0.05 One-to-one session vs. Written material,

a For p < 0.05 One-to-one session vs. Control,

b For p < 0.05 Group session vs. Control,

c For p < 0.05 Written education material vs. Control

## DISCUSSION

No significant differences among groups who received Ramadan specific diabetes education via three different modes were observed. However, patient’s compliance was found to be significantly better among those who received education via any mode compared to those did not receive education.

Studies consistently documented the beneficial effect of patient’s education on safer fasting. Study conducted in 2012, reported the composite effect of alternation of drug dosage and timing, dietary counseling and patient education in the occurrence of acute complications during fasting.^9^

Recently published guidelines on Ramadan and Diabetes has also emphasized imparting Ramadan Specific Diabetes Education to the diabetic subjects.^10^ However; despite the documented benefits and recommendations of education to the patients with diabetes, there is no preferred diabetes education approach which exit.

Patients in the education groups were given Ramadan Specific diabetes education via three different modes. However due to scarcity of data about strategy of delivering education to the patients with diabetes in general and Ramadan specific diabetes education in particular there were limited opportunities to compare the results of present study. We ensured delivery of similar information to all the patients in the education arm.

### Alteration of Drug Dosage and Timing

Literature has constantly documented that fasting in Ramadan is different from starvation largely due to its regular pattern of fasting and eating, type of food consumed, use of medications and daily life styles. Change in the frequency and timing of meals in Ramadan result in mismatch with drug dose and timings.^11^ In 2004, EPIDIAR reported that in 79% and 75% patients with type-1 and type-2 diabetes respectively, oral anti diabetic drug doses remained unchanged; whereas, insulin dose was continued unchanged in 64% of the patients with diabetes (both type-1 and type-2 diabetes) also high rate of complication are shown in that study.^3^ However, a study in 2012 reported that majority (80.8%) of the patients with diabetes, who intend to fast, visit their physicians before Ramadan for adjustment of dose and timing of diabetes medicines. To ensure safer fasting, author(s) identified three areas where diabetes education may be more beneficial. These areas are along with adjustment of dose and timing of medicine monitoring of blood glucose and readiness to break the fast when experienced symptoms of hypoglycemia or hyperglycemia.^12^

Participants who received education were compliant to the change of their drug dosage and timing while only 30% of the patients who did not receive education changed their drug dosage and timing. There was no significant difference among groups who received education highlighting the importance of imparting education, regardless of mode of delivery.

### Self-Monitoring of Blood Glucose

Monitoring of blood glucose during Ramadan is essential for patients with diabetes who hold fast during Ramadan and more particularly in patients with type-1 diabetes and also in patients with type-2 diabetes who require insulin.^13^

One of the largest epidemiological surveys on patient with diabetes (EPIDIAR study) reported that blood glucose monitoring was done by only 67% and 37% of the patients with type-1 type-2 diabetes respectively during Ramadan. While only 17% of the patients checked their blood glucose level even with hypoglycemia and hyperglycemia episodes.^3^ Beneficial effect of patient counseling on monitoring of blood glucose was reported in a prospectively conducted study in nine different diabetes specialist clinics all over Pakistan. Study reported that all the patients with type-1 checked their blood glucose level when they developed hyper or hypo glycemic symptoms whereas 71.43% and 78% patients with type-2 diabetes checked their blood glucose level when they developed hypo and hyper glycemic symptoms respectively during Ramadan.^7^ In the present study majority (>90%) of patients who received education monitored their blood glucose level. This is in contrast to 68.5% of participants in the control group.

### Readiness to break the fast

It was observed from the studies conducted in the past that higher number of patients observed fast end up developing acute diabetes complications.^14^ This may possibly be due to misconception that checking blood glucose during fasting can break the fast. A study at primary care diabetic center in Karachi reported more than 50% of patients and one third of general practitioners (GPs) believe that checking blood glucose break the fast.^15^ Education in the present study succeeded in rectifying this misconception resulted in significantly increased number of patients performed SMBG in the education arm. Patients in the present study were educated about how, when and how often they should check their blood glucose level. This information was supplemented with the knowledge about the symptoms of hypo and hyperglycemia to enable the patient to take the decision of breaking or continuing the fast.

### Appreciation of Symptoms of Hypoglycemia

There was significantly higher appreciation of symptoms of hypoglycemia in the groups who received education contrast to non-education group. Possible explanation may lie in increased awareness of these patients to the hypoglycemia symptoms and their readiness to check the blood glucose level when became symptomatic.

### Appreciation of Symptoms of Hyperglycemia

Diabetes is a hyperglycemic condition. Diabetic patient before diagnosis of this condition may live in this state for a varying period of time. During this period patient may have experienced and intrinsically learnt its symptoms. In the present study, therefore, symptoms of hyperglycemia were appreciated in nearly equal frequency by the patients received as well as those did not receive education. Moreover, in response to appreciation of these symptoms patients in both groups checked their blood glucose level.

### Avoidance of strenuous physical activity during Ramadan

Data regarding physical activities in fasting patients with diabetes during Ramadan is scarce. No guidelines explain the amount of physical activity a diabetic person should undertake during fast. It is however emphasized that light to moderate levels of physical activity on a regular basis during fasting is harmless for patients with diabetes.^16^ Thus normal physical activity to carry out routine life activity may be continued. However, excessive physical activity should be avoided particularly few hours before sunset meal as it may lead to an increased risk of hypoglycemia.^13^ In this study 78-80% of patients received education via different modes keeps themselves abstain from doing moderate/ severe levels of physical activity. The same was also observed in non-education group where 75% of patients keep themselves away from performing the strenuous physical work.

### Limitations

Patients in the education group were inducted before Ramadan and monitored prospectively whereas induction and collection of information from control group was carried out after Ramadan. Thus potential of recall bias may be present that demands careful interpretation. Moreover small number of the study participants and their induction from the OPD of a diabetes institute located in an urban area restrict generalizibility of the findings.

## CONCLUSIONS

Ramadan specific diabetes education (irrespective of mode of delivery) is mandatory to follow the recommendations of Ramadan specific guidelines. Further large scale studies are needed to validate our findings.
